# Modulatory Role of Silver Nanoparticles and Mesenchymal Stem Cell–Derived Exosome-Modified Barrier Membrane on Macrophages and Osteogenesis

**DOI:** 10.3389/fchem.2021.699802

**Published:** 2021-08-02

**Authors:** Haiping Lu, Yi Zhang, Shan Xiong, Yinghong Zhou, Lan Xiao, Yaping Ma, Yin Xiao, Xin Wang

**Affiliations:** ^1^ Department of Orthopaedic Surgery, Affiliated Hospital of Zunyi Medical University, Zunyi, China; ^2^ Department of Hygiene Toxicology, School of Public Health, Zunyi Medical University, Zunyi, China; ^3^ Institute of Health and Biomedical Innovation, Queensland University of Technology, Brisbane, QLD, Australia; ^4^ The Australia−China Centre for Tissue Engineering and Regenerative Medicine (ACCTERM), Brisbane, QLD, Australia

**Keywords:** exosome, mesenchymal stem cells, Ag, PCL hybrid scaffold, osteogenesis

## Abstract

**Background:** As a wound dressing and barrier membrane, surface modification of polycaprolactone (PCL) is needed in order to achieve better biological activities. Exosomes derived from mesenchymal stem cells (MSCs) hold significant tissue regeneration promise. Silver nanoparticles (Ag) have been suggested as the surface modification technique for various medical devices.

**Materials and Methods:** Ag and human bone marrow MSC (hBMSC)-derived exosomes (MSCs-exo) were used to modify the PCL scaffold. The impact of different scaffolds on immune cells and MSC proliferation and differentiation was further evaluated.

**Results:** MSCs-exo exhibited cup-shaped morphology with a diameter around 100 nm. MSCs-exo were enriched with exosome marker CD81 and showed good internalization into recipient cells. 200 ng/ml Ag nanoparticles and MSCs-exo were further used to modify the PCL scaffold. The internalization study further indicated a similar releasing pattern of exosomes from Ag/MSCs-exo hybrid scaffolds into RAW264.7 and hBMSCs at 12 and 24 h, respectively. Macrophages play an important role during different stages of bone regeneration. The MTT and confocal microscopy study demonstrated no significant toxicity of exosome and/or Ag hybrid scaffolds for macrophages and MSCs. Inflammatory macrophages were further used to mimic the inflammatory environment. A mixed population of elongated and round morphology was noted in the exosome and Ag hybrid group, in which the proinflammatory genes and secretion of IL-6 and TNF-α were significantly reduced. In addition, the exosome and Ag hybrid scaffolds could significantly boost the osteogenic differentiation of hBMSCs.

**Discussion:** This study highlights the possibility of using Ag nanoparticles and MSCs-exo to modify the PCL scaffold, thus providing new insight into the development of the novel immunomodulatory biomembrane.

## Introduction

The incident of bone injury has been increased due to the aging of our society and changing of our lifestyle, such as increased obesity and poor levels of physical activity (Liang and Chikritzhs, 2016). Despite the intrinsic regenerative ability of the bone and advancement in treatment approaches, about 5–10% of patients still display compromised bone healing under various physiological and pathological conditions (Buza and Einhorn, 2016). To date, autografts remain the gold standard for treating large defects due to their inherent autoinduction and biocompatible properties. However, the source is very limited, and the surgical procedure may cause further injury to the patients (Baldwin et al., 2019). To solve these problems, synthesized bone-substitutional materials have been developed, whereas the drawbacks of the current materials, such as low osteoinductivity and triggering inflammatory response, limit the translation use, suggesting that strategies should be developed to improve the current bone-substitutional biomaterials.

Exosomes are nanosized membrane-bound extracellular vesicles secreted by most of the eukaryotic cells, which play an important role in intercellular communication (Kalluri and LeBleu, 2020). Exosomes are important nanocarriers for transferring several signaling molecules, such as proteins, lipids, endogenous genetic information, and growth factors, from parent cells to recipient cells (Luan et al., 2017). Exosome-derived therapy holds significant clinical translational potential due to its unique regulatory and therapeutic properties and minimized complications due to noncell involvement. MSCs are multipotent cells with numerous therapeutic potentials for regenerative medicine (Parekkadan and Milwid, 2010). Previous studies indicated a possible paracrine mechanism of MSC-based therapy, in which multiple factors secreted from MSCs act as important regulators in tissue regeneration. Apart from growth factors and cytokines (Joseph et al., 2020), exosomes from MSCs have been demonstrated as potential therapeutic agents under various physiological and pathological conditions (Hu et al., 2019). The tissue regenerative capacity of MSC-exo in bone regeneration has been found critical in the modulation of early bone regeneration after injury as well as in regulating the immune response and angiogenesis (Qin et al., 2016; Liu et al., 2019). One of the studies found that MSC-derived exosomes and conditioned medium from MSCs enhanced bone regeneration and angiogenesis in the rat bone defect model, indicating the important regenerative ability of MSC-derived exosomes on bone regeneration (Takeuchi et al., 2019). Animal studies also indicated that irradiation-induced bone loss can be protected by an intravenous injection of MSC-derived exosomes, in which the oxidative stress and DNA damage in the recipient cells can be significantly reduced following exosome treatment (Zuo et al., 2019). In addition, exosome secreted by MSCs has been shown to effectively repair the damages of myocardial infarction (Teng et al., 2015), liver disease (Lou et al., 2017), kidney injury, lung injury (Lee et al., 2012), etc. These data are strong indications that MSC-derived exosomes have promising potential for bone tissue regeneration.

Silver ions have been used as broad-spectrum bactericides owing to their bactericidal activity for thousands of years (Silvestry-Rodriguez et al., 2007). Ag nanoparticles have gained huge importance over the past years for medical purposes as antimicrobial and anticancer agents (Zhang et al., 2016a). However, the physical and chemical properties of Ag nanoparticles, especially particle surface area, surface chemistry, particle size, particle morphology, and composition, are important factors to influence their biological activity, toxicity, and biodegradability (Zhang et al., 2016a). Additionally, surface immobilization using Ag nanoparticles has been frequently used in various antibacterial implants, such as wound dressings and catheters (Besinis et al., 2017; Choi et al., 2019). Recently, the immunoregulatory role of Ag nanoparticle-modified surface on immune cells has also been reported (Chen et al., 2020). PCL, a high-performance bioresorbable and biocompatible polymer, has been approved by the US Food and Drug Administration (FDA) for soft and hard tissue repair (Mondal et al., 2016). However, the PCL scaffold is relatively bio-inert, resulting in an unsatisfactory therapeutic effect. Therefore, in this work, we sought to generate PCL-based hybrid scaffold with incorporation of MSC-derived exosomes and Ag nanoparticles to improve osteogenesis and bone regeneration. Our results indicate that the novel exosomes and Ag hybrid scaffold might offer unique immunomodulation on osteogenesis, thus serving as a potential therapeutic scaffold for hard tissue regeneration.

## Materials and Methods

### Reagents

Alexa Fluor™ 594-labeled phalloidin (A12381) and Pierce™ BCA Protein Assay Kit (23227) were purchased from Thermo Fisher Scientific, China. 4′,6-Diamidino-2-phenylindole dihydrochloride (DAPI, D9542), PKH67 Green Fluorescent Cell Linker Kit for General Cell Membrane Labeling (MINI67-1KT), lipopolysaccharide (LPS, *Escherichia coli* 0111: B4, L4391), β‐glycerophosphate (G9422), L‐ascorbic acid 2‐phosphate (49752), and dexamethasone (D1756) were from Sigma, China. Antibodies used in this study were rabbit polyclonal antibody against alkaline phosphatase (ALP) (ab126820; Abcam, China) and CD81 antibody (B-11) (sc-166029; Santa Cruz Biotechnology, China).

### Cell Culture and Exosome Isolation

The human bone marrow–derived mesenchymal stromal cells (hBMSCs, ATCC® PCS-500-012™) were used for exosome’s isolation. Cells were maintained in Dulbecco’s Modified Eagle Medium (DMEM; Thermo Fisher Scientific, China) supplemented with 10% fetal bovine serum (FBS; Biological Industries Ltd., Beit Haemek, Israel) and 1% (v/v) penicillin/streptomycin (Solarbio, Beijing, China). For conditioned medium (CM) collection, hBMSCs (within passage 3) were seeded in T75 flasks at a density of 5 × 10^5^/well overnight at 37°C. After rinsing three times with phosphate-buffered saline (PBS), the cells were cultured with 10 ml DMEM supplemented with 10% exosome-depleted FBS at 37°C for 24 h prior to the CM collection for exosome isolation. Exosomes were isolated according to published guidelines (Théry et al., 2018). First, the CM was filtered through 0.22 µm filters to remove live cells and other large membranous structures. Then, the CM was centrifuged at 300 × g and 4°C for 10 min to pellet any remaining live cells. Second, the CM was transferred to new tubes and centrifuged at 2,000 × g and 4°C for 20 min. Then, the CM was centrifuged in a 41 Ti rotor (Beckman) at 10,000 × g at 4°C for 40 min. After carefully transferring the supernatant into a new tube, samples were spun at 100,000 × g and 4°C for 90 min. The exosome pellets were resuspended in PBS, aliquoted, and stored at −80°C for future use.

The murine-derived macrophage cell line RAW264.7 cells (ATCC® TIB-71™) were maintained in Dulbecco’s Modified Eagle Medium (DMEM; Thermo Fisher Scientific, China) supplemented with 10% heat-inactivated fetal bovine serum (FBS; Biological Industries Ltd., Beit Haemek, Israel) and 1% (v/v) penicillin/streptomycin (Solarbio, Beijing, China) in a humidified incubator containing 5% CO_2_ at 37°C.

### Characterization of MSC-Derived Exosomes

To visualize MSC-derived exosomes, transmission electron microscopy (TEM) was used. In brief, 5 μl exosome was used for sample preparation. Carbon/formvar-coated copper grids were placed on 5 μl exosomes for 5 min at room temperature. After removing excess solution, the grids were stained with 1% uranyl acetate for 20 s. After rinsing with deionized water, the TEM grids were completely dried using Whatman filter paper. Images were taken using JEM-1400, JEOL, Japan, at 80 kV.

To identify the exosomal marker, exosomes (20 µg in terms of protein) were lysed using RIPA lysis buffer supplemented with a protease inhibitor cocktail (04693132001; Roche Diagnostics, Indianapolis, IN, United States). Western blotting was performed according to our previous published protocol (Xiao et al., 2020). In brief, proteins were separated using 4–12% gradient Bolt™ Bis-Tris Plus Gel according to the manufacturer’s instructions (Thermo Fisher Scientific, China). After blocking with Odyssey buffer, the blots were incubated with CD81 (1:200) overnight at 4°C. The blots were incubated with IRDye^®^ 680RD goat anti-mouse IgG (H+L) (1:10,000; LI-COR Biotechnology, United States) and washed thrice with PBS Tween 20 (0.1%). Protein signals were visualized using Odyssey Infrared Imaging System (LI-COR Biotechnology, United States).

### Exosome Internalization into Cells

To evaluate exosome internalization into cells, exosome labeling was performed using PKH67 Green Fluorescent Cell Linker Kit according to the manufacturer’s instruction. In brief, exosomal protein quantification was performed using the Pierce BCA Protein Assay Kit. Next, exosomes (10 µg in terms of protein) were stained with PKH67 solution for 3 min at room temperature before adding DMEM supplemented with 1% FBS (exosome-depleted) for 1 min to terminate the staining process. PKH67-labeled exosomes were incubated with recipient cells at 37 °C. Cells were fixed with 4% paraformaldehyde (PFA, P6148; Sigma, United States) and stained with Alexa Fluor 594-labeled phalloidin. Images were captured using a confocal laser scanning microscope with a ×40 objective (Leica DM IRB; Leica, Wetzlar, Germany).

### MTT and Live/Dead Assay

MTT cell proliferation assay was performed according to the manufacturer’s instructions. In brief, 20 μl of 5 mg/ml MTT (3-(4, 5-dimethylthiazol-2-yl)-2, 5-diphenyl tetrazolium bromide) (M2128; Sigma, China) was added into each well, and plates were incubated for additional 4 h at 37°C in a CO_2_ incubator. After carefully removing the supernatant, 100 µl dimethyl sulfoxide (DMSO) was used in each well until the formazan crystals have been dissolved. The absorbance of the sample was measured using a microplate reader at 570 nm.

Live/dead staining was performed according to the previous study (Herzfeld et al., 2014). In brief, cells were incubated with staining solution containing 5 mg/ml FDA (fluorescein diacetate, green) and 2 mg/ml PI (propidium iodide, red) at room temperature for 4 min in the dark. After washing with PBS, samples were prepared for observation using an inverted fluorescence microscope with a ×10 objective (Leica, Wetzlar, Germany).

### Scaffold Fabrication and Modification

PCL scaffolds were prepared by electrospinning. In brief, 10% (w/v) PCL solution was prepared by dissolving PCL in 1,1,1,3,3,3-hexafluoro-2-propanol (HFP) (Sigma, China). The solution was loaded into a syringe and fed through a syringe pump at a flow rate of 2 ml/h. The needle was connected to a high voltage of 20 kV at a distance of 20 cm. The PCL scaffolds were dried in vacuum to evaporate the residuals at room temperature. Poly(dopamine) (PDA) coating was used to initially modify the PCL surface (Li et al., 2020). PCL scaffolds were immersed into 4 mg/ml dopamine hydrochloride in 10 mM pH = 8.5 Tris buffer for 1 h with stirring before rinsing with Milli-Q water. After coating, the PCL scaffolds were regarded as PCL/PDA scaffolds. Silver nanoparticles (Ag, 40 nm diameter) were purchased from Sigma (730807, China). PCL/PDA scaffolds were then incubated with Ag (200 ng/ml) overnight to fabricate the PCL/PDA+Ag scaffold. For exosome coating, exosomes were incubated with the PCL/PDA or PCL/PDA+Ag scaffold for 1 h at room temperature.

### Inflammatory Macrophage Response to Different Scaffolds

To determine the effect of the inflammatory macrophage response to different scaffolds, coculture study was performed. In brief, macrophages were seeded on the coverslip at a density of 1 × 10^5^ overnight at 37°C before stimulating with 1000 ng/ml of LPS. After 12 h stimulation, LPS was removed, and cells were rinsed three times with PBS before coculturing with different scaffolds. After 24 h incubation, cells were fixed by 4% PFA and stained with Alexa Fluor 594-labeled phalloidin. Cells were photographed using an inverted confocal microscope (Leica DM IRB; Leica, Wetzlar, Germany).

### Osteogenic Differentiation of hBMSCs

For alkaline phosphatase (ALP) immunofluorescence staining, hBMSCs were seeded at a density of 1 × 10^5^/well overnight at 37°C. After treatment, cells were washed once with PBS, fixed by 4% PFA for 10 min at room temperature, then permeabilized with 0.25% Triton X-100 (T8787; Sigma, China) for 10 min, and blocked with 4% bovine serum albumin (BSA) for 1 h at room temperature. ALP staining was performed using the rabbit polyclonal antibody to ALP antibody (1:100) overnight at 4°C. Fluorescein isothiocyanate-conjugated goat anti-rabbit IgG (1:1000) was used as a secondary antibody. Actin was stained with Alexa Fluor 594-labeled phalloidin. All images were taken using a confocal microscope (Leica DM IRB; Leica, Wetzlar, Germany) with a 40× oil immersion lens, laser wavelength with excitation and emission wavelengths of 488 and 520 nm, respectively, and 1024 × 1024 resolution.

For the ALP staining assay, hBMSCs were seeded at a density of 1 × 10^5^/well overnight at 37°C. After treatment, cells were washed by 300 µl wash buffer and stained with ALP staining solution for 30 min at 37°C. After rinsing twice with wash buffer, images were taken using an inverted light microscope.

ALP activity was determined using Alkaline Phosphatase Assay Kit according to the manufacture’s instruction (ab83369; Abcam, China).

### Real-Time Quantitative Reverse Transcription Polymerase Chain Reaction (RT-PCR)

hBMSCs were seeded in six-well plates at a density of 1 × 10^5^/well overnight at 37°C. After treatment, cells were harvested for RNA extraction using TRIzol reagent (15596026; Thermo Fisher Scientific, China) according to the manufacturer’s instructions. RNA concentration was measured using the NanoDrop 8000 spectrophotometer (NanoDrop Technologies). cDNA synthesis was performed using the RevertAid First Strand cDNA Synthesis Kit (Thermo Fisher Scientific, China) according to the manufacturer’s instruction. 500 ng of total RNA was used as the template for reverse transcription. RT-PCR was performed using SYBR Green qPCR Master Mix (Life Technologies, China) on an ABI Prism 7500 Thermal Cycler (Applied Biosystems, Foster City, CA, United States). All primer sequences were analyzed for target specificity on Primer-BLAST and were purchased from Sigma, China. The primer sequences are listed in Table 1. The difference between the mean Ct values of the gene of interest and the housekeeping gene was labeled ΔCt, and the relative expression was calculated using the comparative Ct (2^−ΔΔCT^) method (Schmittgen and Livak, 2008).

**TABLE 1:** Primers used in RT-PCR.

Genes	Primer sequences
IL-6	Forward: 5′-ATAGTCCTTCCTACCCCAATTTCC-3′ Reverse: 5′-GATGAATTGGATGGTCTTGGTCC-3′
TNF-α	Forward: 5′-CTGAACTTCGGGGTGATCGG-3′ Reverse: 5′-GGCTTGTCACTCGAATTTTGAGA-3′
iNOS	Forward: 5′-CGAAACGCTTCACTTCCAA-3′ Reverse: 5′- TGAGCCTATATTGCTGTGGCT-3′
IL-1β	Forward: 5′-TGGAGAGTGTGGATCCCAAG -3′ Reverse: 5′-GGTGCTGATGTACCAGTTGG -3′
ALP	Forward: 5′-CGTGGCTAAGAATGTCATCATGTT-3′ Reverse: 5′-TGGTGGAGCTGACCCTTGA-3′
Col-I	Forward: 5′-CCCTGGAAAGAATGGAGATGAT-3′ Reverse: 5′-ACCATCCAAACCACTGAAACCT-3′
Runx2	Forward: 5′-CATGGCGGGTAACGATGAA-3′ Reverse: 5′-AGACGGTTATGGTCAAGGTGAAA-3′
OPN	Forward:5′- GACCAAGGAAAACTCACTAC-3′ Reverse: 5′- CTGTTTAACTGGTATGGCAC-3′
GAPDH	Forward: 5′-TCAGCAATGCCTCCTGCAC -3′ Reverse: 5′-TCTGGGTGGCAGTGATGGC -3′

### Statistical Analysis

All the data were expressed as mean ± standard deviation (SD, *n* = 3). Statistical analysis was performed using GraphPad Prism 7 (version 7.02) for Windows (GraphPad Software Inc., United States). Statistical differences between groups were determined with one-way analysis of variance (ANOVA) with Bonferroni’s multiple comparison tests. A value of *p* < 0.05 was considered statistically significant.

## Results and Discussion

### Characterization of MSC-Derived Exosomes

**FIGURE 1:**
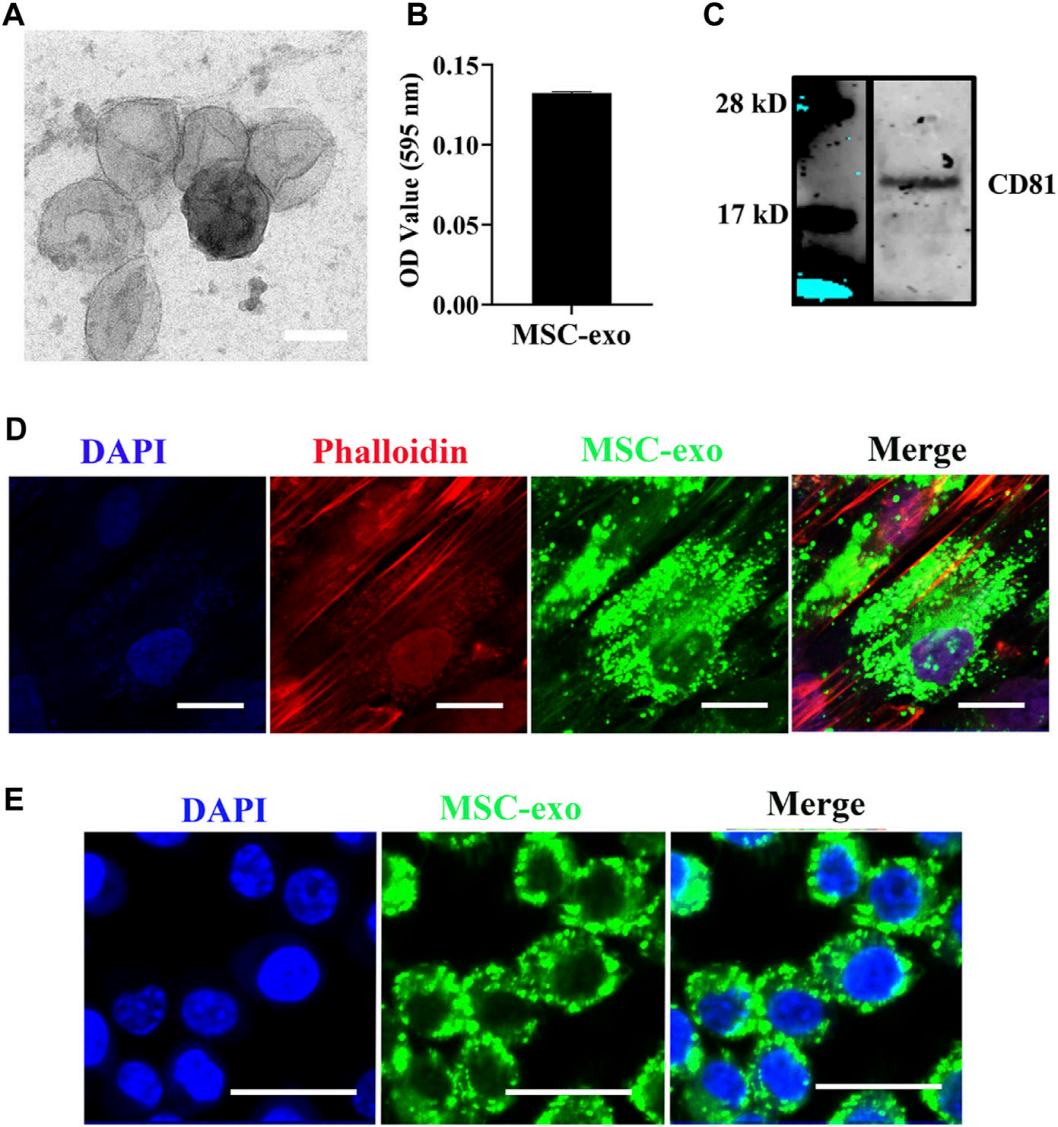
hBMSC-derived exosome isolation and characterization. **(A)** Representative TEM images of exosomes isolated from MSCs. Exosomes were negatively stained with 1% uranyl acetate and examined by TEM. Scale bars = 80 nm. **(B)** Quantification of the exosomal protein level. Protein quantification was assessed using the Pierce BCA Protein Assay. Data were expressed as mean ± SD (*n* = 3). **(C)** Western blot of the exosomal surface marker. Expression of CD81 was evaluated by western blotting. **(D)** Internalization of hBMSC-derived exosomes into hBMSCs. hBMSC-derived exosomes were labeled with the exosome labeling marker, PKH67, and then incubated with hBMSCs overnight at 37°C. Cells were stained with the cytoskeleton (red) and nucleus (blue) (*n* = 3). Scale bars = 20 μm. **(E)** Internalization of hBMSC-derived exosomes into macrophages. hBMSC-derived exosomes were labeled with the exosome labeling marker, PKH67, and then incubated with RAW264.7 overnight at 37°C. Scale bars = 20 μm.

In the field of tissue regeneration, MSC-derived exosomes present promising therapeutic potential due to their unique regenerative abilities (Liu et al., 2019). The isolated exosomes were first characterized by TEM. As shown in Figure 1A **,** cup-shaped morphology was observed from the isolated exosomes. The results indicated that the diameter of isolated exosomes is about 100 nm. The morphology of hBMSC-derived exosomes has been described previously, and hBMSC-derived exosomes showed cup-shape morphology with an average size from 50 to 80 nm (Wang et al., 2014a). In addition, Ni et al. demonstrated that membrane vesicles isolated from the rodent bone marrow exhibit similar sizes ranging from 30 to 150 nm (Ni et al., 2019). In addition, we next quantified the total amount of proteins from isolated exosomes. The average protein concentration of MSC-derived exosomes is 1.66 μg/μl (Figure 1B). We next used western blotting to examine the exosomal marker (CD81). The result showed that isolated exosomes expressed significant levels of CD81 (Figure 1C). Internalization of exosomes into recipient cells is mediated by direct fusion of the plasma membrane or receptor-mediated endocytosis, transferring important modulatory proteins or genetic materials into recipient cells (Delenclos et al., 2017). To examine the cellular internalization of isolated exosomes into hBSMCs, exosome labeling marker PKH carbocyanine dye (Puzar Dominkus et al., 2018) was used to incubate hBMSCs for 12 h. Confocal microscopy was used for analysis. As shown in Figure 1D, PKH67-labeled exosomes were observed around perinuclear areas, indicating good internalization of MSC-derived exosomes into recipient cells. The exosome uptake by macrophages was further tested by confocal microscopy. As shown in Figure 1E, PKH67-labeled exosomes were also observed around perinuclear areas.

### Characterization of the Exosome and Ag Hybrid Scaffold

**FIGURE 2:**
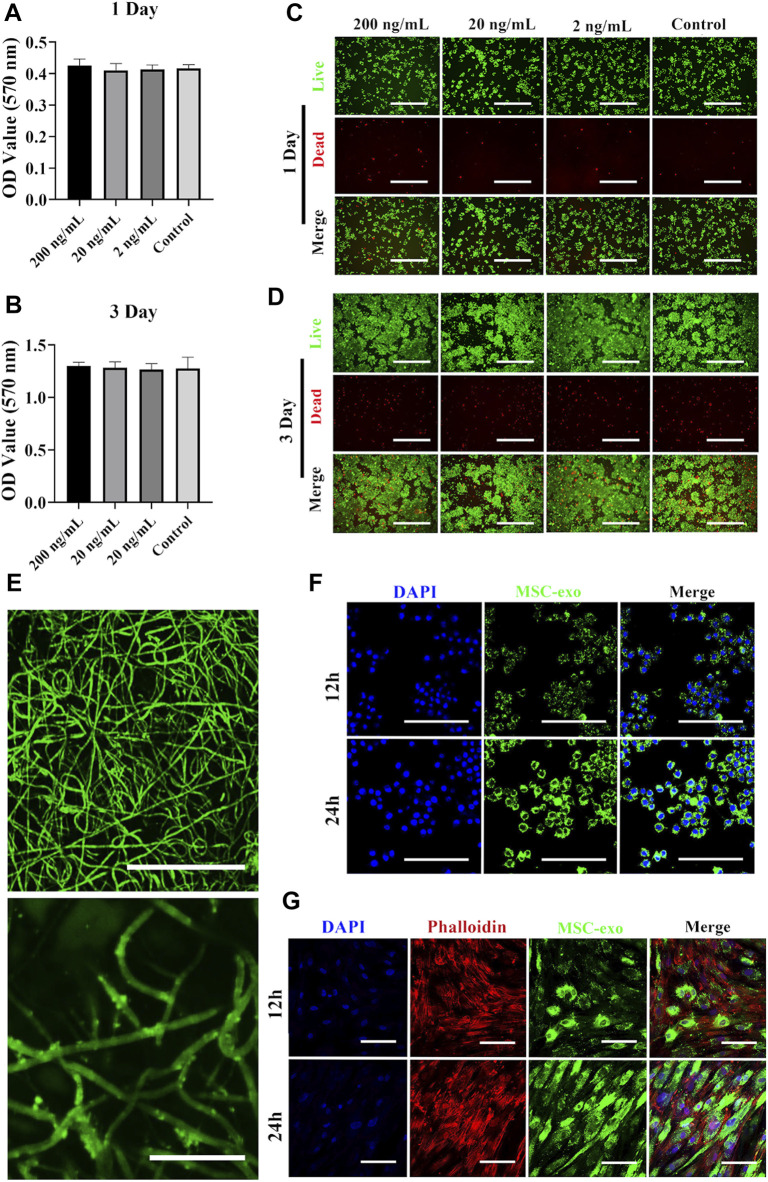
Surface characterization of the exosome and Ag hybrid scaffold. **(A**,**B)** Metabolic activity of macrophages assessed by the MTT assay. RAW264.7 cells were seeded at a density of 5 × 10^3^/well overnight at 37°C. Cell proliferation in the presence or absence of Ag was evaluated by the MTT assay at 1 and 3 days. Error bars denote the mean ± SD (*n* = 5). **(C**,**D)** Live and dead assay of macrophages. RAW264.7 cells were seeded at a density of 5 × 10^3^/well overnight at 37°C. After Ag treatment, the cells were stained with FDA (live/green) and PI (dead/red) and were examined by confocal microscopy. Scale bars = 200 μm. **(E)** Representative confocal microscopy images of the exosome and Ag hybrid scaffold. Exosomes were prelabeled with PKH67 exosome staining solution. Scale bars = 100 μm (top panel). Scale bars = 20 μm (bottom panel). **(F)** Exosome released from the MSC-exosome and Ag hybrid scaffold was uptake by macrophages. Scale bars = 100 μm. **(G)** Exosome released from the MSC-exosome and Ag hybrid scaffold was uptake by hBMSCs. Scale bars = 100 μm. Effect of the exosome and Ag hybrid scaffold on macrophages.

Using the MTT assay, we first tested the viability of RAW264.7 cells when exposed to different concentrations of Ag nanoparticles. As shown in Figures 2A,B, Ag at concentration up to 200 ng/ml caused no significant decrease of viability. FDA and PI were used to label live (green) and dead (red) cells, respectively (Figures 2C,D). Nearly all macrophages were viable when cultured with 200 ng/ml Ag. Despite the low toxicity of Ag at 200 ng/ml (as observed in our study), several studies have tested the viability of different cells exposed to Ag nanoparticles. For example, Park et al. found that RAW264.7 cells exposed to different concentrations of Ag nanoparticles showed a dose- and time-dependent decrease of cell viability (Park et al., 2010a). In another study, RAW264.7 cells were used to test Ag toxicity, and the results indicated that Ag showed no significant toxicity even at high concentrations (Alsaleh et al., 2019). We next fabricated an exosome-based and Ag-guided biomembrane scaffold to regulate the early osteogenesis environment. As shown in Figure 2E, PKH67-labeled green membrane vesicles could be observed on the PCL/PDA+Ag biomembrane, indicating good attachment of MSC-derived exosomes on PCL/PDA+Ag scaffolds. The exosome releasing potential from the hybrid scaffold was examined with coculture assays using hBMSCs and macrophages as recipient cells. To do this, PKH67-labeled exosomes were used to mark the exosome before scaffold prefabrication. As shown in Figures 2F,G, green fluorescence puncta were observed around perinuclear areas after incubating with the Ag/exosome hybrid scaffold. At 12 h, macrophages and hBMSCs showed a similar exosome uptake pattern, indicating good cellular internalization of exosomes released from the Ag/exosome hybrid scaffold.

**FIGURE 3:**
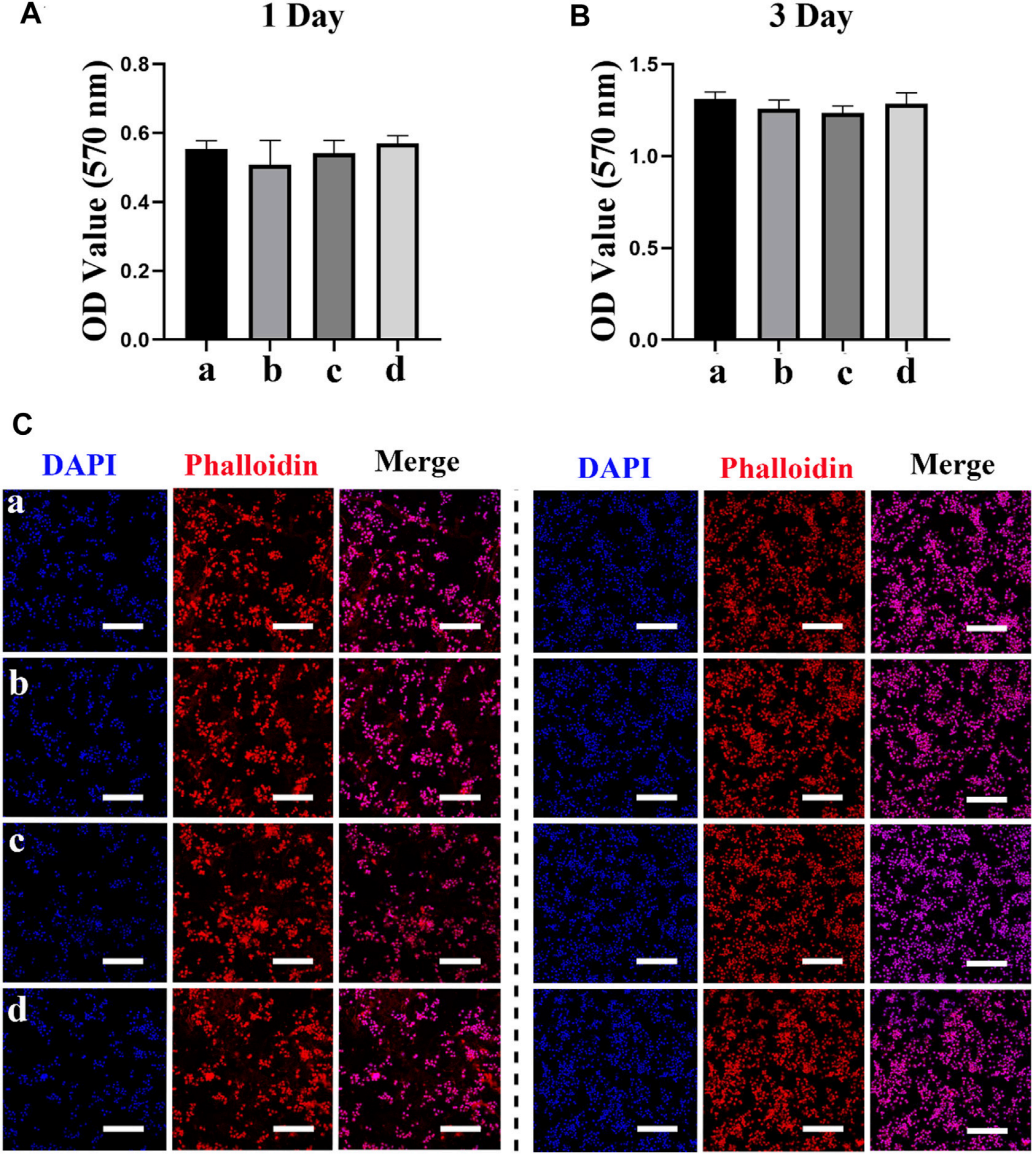
Effect of the exosome and Ag hybrid scaffold on macrophages. **(A,B)** The cellular metabolic activity was determined by the MTT assay. a = PCL/PDA, b = PCL/PDA+Ag, c = PCL/PDA+exosome, and d = PCL/PDA+Ag+exosome. Results are shown as mean ± SD (*n* = 5). **(C,D)** Representative confocal microscopic images of RAW264.7 cocultured with the MSC-exosome and/or Ag hybrid scaffold at 1 day **(C)** and 3 days **(D)**. Cells were stained with Alexa Fluor 594-conjugated phalloidin (red) and nucleus (blue) (*n* = 3). Scale bars = 200 μm. Effect of the exosome and Ag hybrid scaffold on macrophage inflammatory response.

We next evaluated the effects of different scaffolds (PCL/PDA, PCL/PDA+Ag, PCL/PDA+exosome, and PCL/PDA+Ag+exosome) on macrophage proliferation by the MTT assay. The results of the cytotoxicity of different scaffolds to macrophages are presented in Figures 3A,B. The MTT assay results indicated no significant cytotoxicity of exosome and/or Ag hybrid scaffolds. Phalloidin and DAPI were used to label the cytoskeleton and nucleus, respectively. Figures 3C,D show the morphology of RAW264.7 cells after coculture with exosome and/or Ag hybrid scaffolds. The confocal microscopy results indicated the similar growth patterns among different groups.

### Effect of exosome and Ag hybrid scaffold on macrophage inflammatory response

**FIGURE 4:**
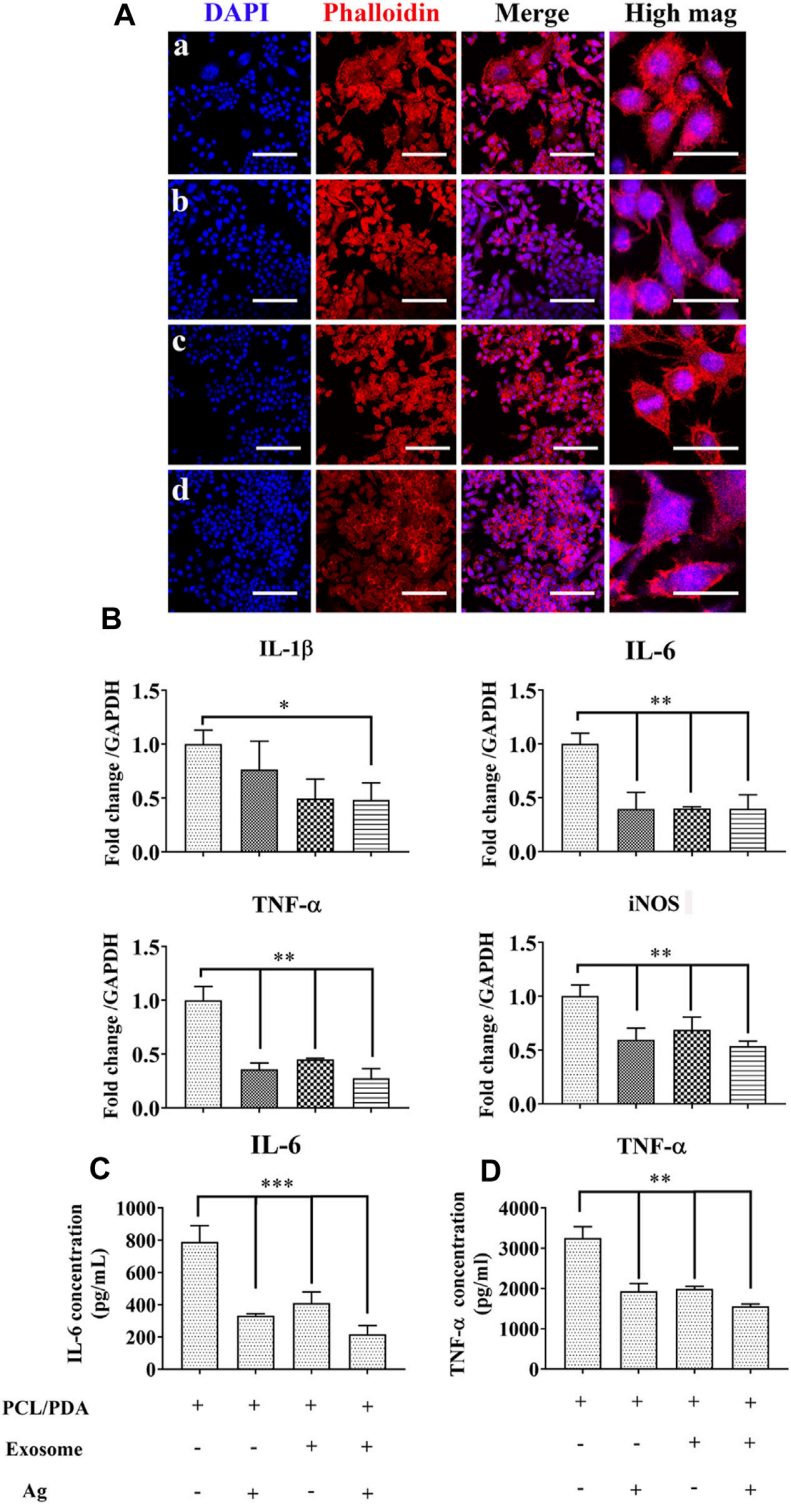
Effect of the exosome and Ag hybrid scaffold on inflammatory macrophages. **(A)** Representative confocal microscopy images of RAW264.7 cells cocultured with the exosome and Ag hybrid scaffold. Inflammatory macrophages were induced by 1000 ng/ml LPS stimulation for 12 h. After washing, inflammatory macrophages were cocultured with the MSC-exosome and Ag hybrid scaffold. Cells were stained by Alexa Fluor 594-conjugated phalloidin and DAPI. (a–d) refer to LPS+PCL/PDA, LPS+PCL/PDA/Ag, LPS+PCL/PDA/exosome, and Ag/exosome hybrid, respectively. Scale bars = 100 μm. Scale bars = 20 μm (high mag). **(B)** Relative expression of inflammation-related genes. RAW264.7 cells were stimulated with 1000 ng/ml LPS for 12 h before coculturing with different scaffolds for another 24 h. The values were normalized with respect to housekeeping gene GAPDH. Error bars denote the mean ± SD. Statistically significant difference (*p < 0.05 and **p < 0.01). **(C)** Expression of IL-6 produced in response to the MSC-exosome and Ag hybrid scaffold under the inflammatory environment. The level of IL-6 was measured using cytokine-specific ELISA. All the data were expressed as mean ± SD (*n* = 3). *** indicates statistically significant difference (*p* < 0.005). **(D)** Expression of TNF-α produced in response to the MSC-exosome and Ag hybrid scaffold under the inflammatory environment. The level of TNF-α was measured using cytokine-specific ELISA. All the data were expressed as mean ± SD (*n* = 3). ** indicates statistically significant difference (*p* < 0.01). Effect of the exosome and Ag hybrid scaffold on hBMSC proliferation.

Initial inflammatory stage is essential for sustaining bone regeneration (Goodman et al., 2019). However, excessive inflammatory conditions are often recorded in conditions such as open fractures and the presence of multiple injuries, thus causing delayed or compromised bone regeneration (Mensah et al., 2009; Takayanagi, 2005; Greenblatt and Shim, 2013; Konstantinidis et al., 2013). In addition, using a critical-sized bone defect model in rats, we also found a significant increase in IL-1β levels in the delayed healing model compared to the normal healing control (Wang et al., 2016). These results suggest that prolonged inflammation is detrimental to successful bone regeneration. We next evaluated the potential regulatory role of different scaffolds on inflammation. We first used confocal microscopy to observe the macrophage morphology. LPS polarizes macrophages toward the inflammatory phenotype, leading to a series of signaling cascade activation and the secretion of proinflammatory cytokines, which is commonly used in studies to simulate inflammation (Wang et al., 2014b). Once polarized, activated macrophages change their morphology; for example, M1-polarized macrophages display a round morphology, while the M2-polarized phenotype shows elongated shapes (McWhorter et al., 2013). The results of the macrophage morphological response to different scaffolds are presented in Figure 4A. Confocal microscopy indicated a mixed population of elongated and pancake-like morphology after coculturing with exosome and/or Ag hybrid scaffolds. We next evaluated the expression of inflammatory genes in macrophages with RT-PCR. As shown in Figure 4B, Ag, exosome, and Ag/exosome hybrid scaffolds significantly decreased the mRNA levels of the inflammatory markers. The inflammatory cytokine levels were further evaluated by ELISA. As shown in Figures 4C,D, the production of IL-6 and TNF-α was significantly reduced in Ag-modified, exosome-modified, and Ag/exosome hybrid scaffolds. Our results echo previous studies examining the role of hBMSC-derived exosomes and Ag in modulating inflammation. For example, Ag nanoparticles demonstrated anti-inflammatory effects by reducing LPS-induced reactive oxygen species generation and TNF-α production (Gonzalez-Carter et al., 2017). In addition, Ag nanoparticles can also attenuate airway inflammation in the murine allergic airway disease model (Park et al., 2010b). These results are clear indication that Ag nanoparticles could serve as important regulators for the inflammatory condition. Exosomes from MSCs also hold important inflammation-regulatory ability, such as reducing myocardial ischemia–reperfusion injury (Zhao et al., 2019), restoring renal injury and dysfunction (Nargesi et al., 2017), and ameliorating lung injury (Aslam et al., 2009). Therefore, these Ag/exosome hybrid scaffolds may show synergistic inflammation-regulatory ability.

### Effect of exosome and Ag hybrid scaffold on hBMSCs proliferation

**FIGURE 5:**
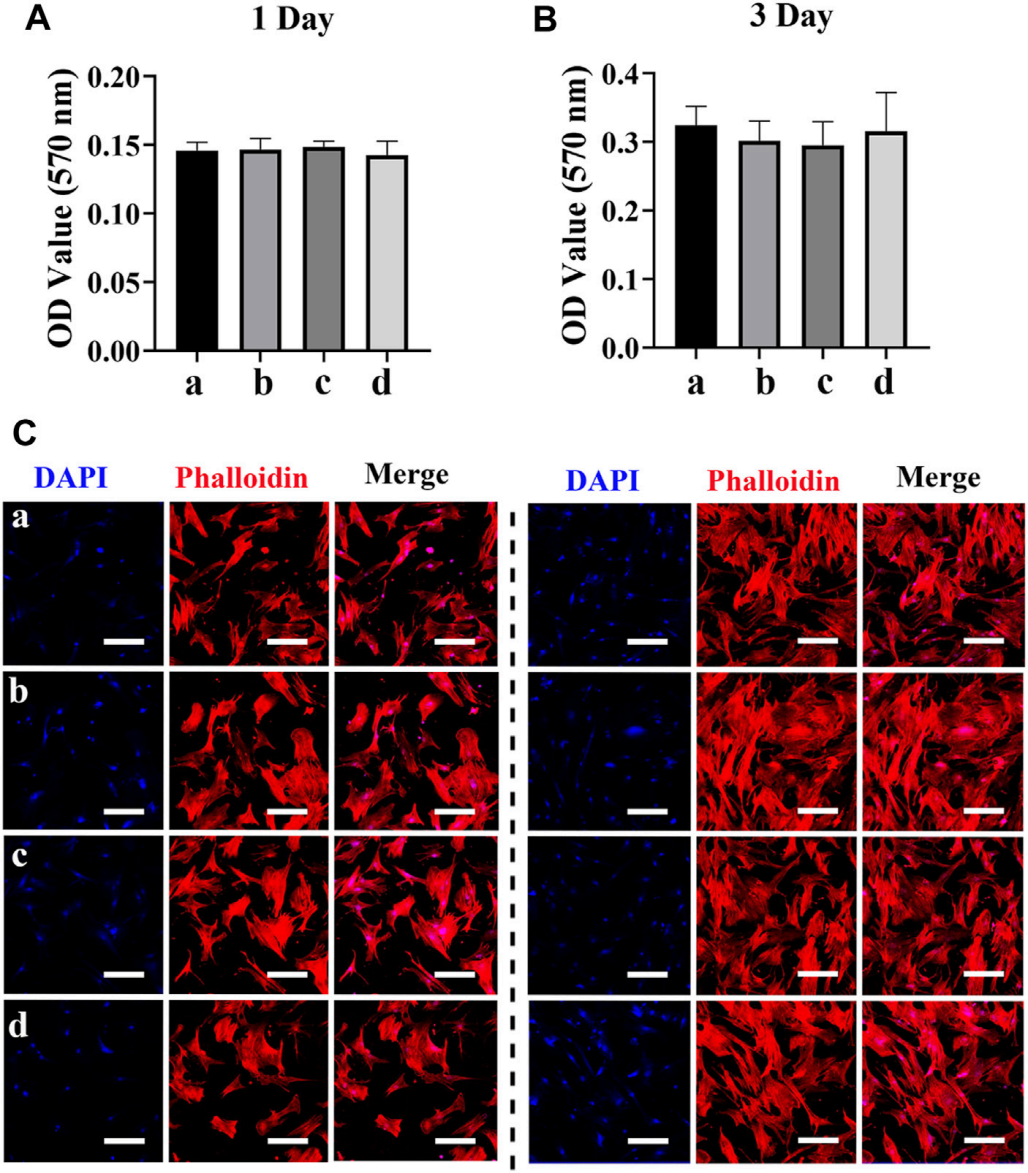
Effect of the exosome and Ag hybrid scaffold on hBMSC proliferation. **(A,B)** Metabolic activity of hBMSCs was assessed by the MTT assay. Cell proliferation was evaluated by the MTT assay at 1 and 3 days. a = PCL/PDA, b = PCL/PDA+Ag, c = PCL/PDA+exosome, and d = PCL/PDA+Ag+exosome. Data were presented as mean ± SD (*n* = 5). **(C,D)** Representative confocal microscopy images of hBMSCs at 1 day **(C)** and 3 days **(D)**. Cells were stained by Alexa Fluor 594-conjugated phalloidin and DAPI and were examined by confocal microscopy. Scale bars = 200 μm. Effect of the exosome and Ag hybrid scaffold on hBMSC osteogenic differentiation.

MSCs are multipotent cells with the regenerative ability to repair various tissue injuries, including skeletal damages (Wu et al., 2014). We investigated the effect of the Ag/exosome hybrid scaffold on MSC response. The potential toxicity of different scaffolds on hBMSCs was evaluated with the MTT assay. As shown in Figures 5A,B, we found no significant differences on MSC proliferation when hBSMCs were cultured with or without the hybrid scaffolds. The effect of Ag nanoparticles on MSC proliferation has been investigated previously. It has been reported that Ag nanoparticles at concentrations of 2.5, 5, and 10 μg/ml showed no significant cytotoxic effects on hBMSCs after 14 days of incubation (Sengstock et al., 2014), which is in accordance with our current study. We then performed phalloidin and DAPI staining to evaluate cell morphology after coculturing with or without the hybrid scaffolds. Figures 5C,D show the morphology of hBMSCs after coculturing with exosome and/or Ag hybrid scaffolds. hBMSCs showed similar growth patterns and good spreading, indicating no potential toxicity. The analysis of MSC proliferation and morphology was in agreement with that of macrophages, which indicated good biocompatibility of the Ag/exosome hybrid scaffold.

**FIGURE 6:**
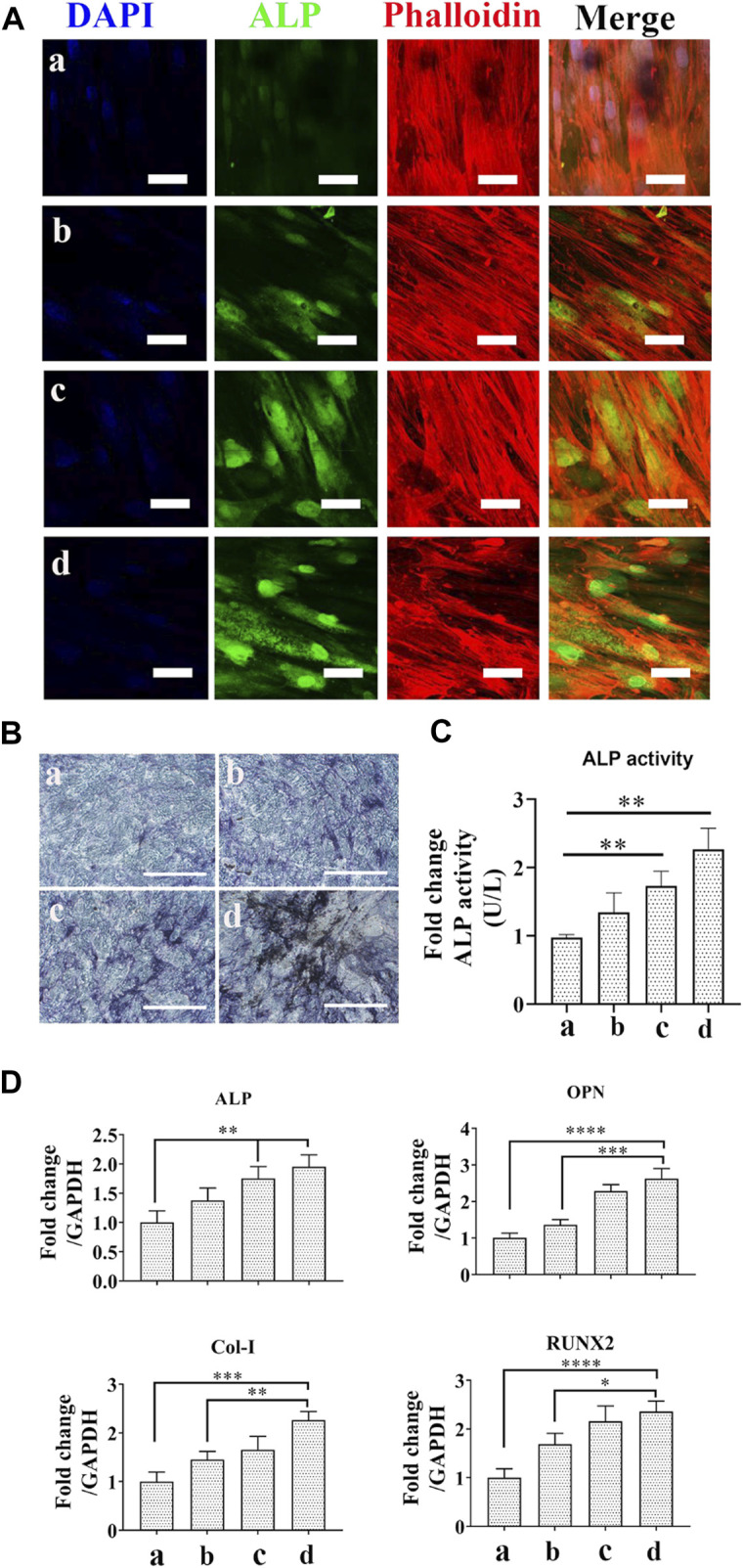
Effect of the exosome and Ag hybrid scaffold on hBMSC differentiation. **(A)** Representative CLSM images of ALP expression. a = PCL/PDA, b = PCL/PDA+Ag, c = PCL/PDA+exosome, and d = PCL/PDA+Ag+exosome. The cytoskeleton was stained with phalloidin (red), and nuclei were stained with DAPI (blue). Representative CLSM images are shown in the figure (*n* = 3). Scale bars = 50 μm. **(B)** Representative images of ALP staining. ALP activity was examined by ALP staining. Scale bars = 200 μm. **(C)** ALP activity. ALP activity was measured using Alkaline Phosphatase Assay Kit. Error bars denote the mean ± SD (*n* = 3). ** indicates significant difference (*p* < 0.01). **(D)** Relative expression of osteogenesis-related genes. Error bars denote the mean ± SD (*n* = 3). Significant differences (**p < 0.01, ***p < 0.005, and ****p < 0.001).

To investigate the effect of different scaffolds on osteogenic differentiation of hBMSCs, cells were cocultured with Ag and/or exosome hybrid scaffolds. As one of the critical markers for osteogenic differentiation, we first evaluated the expression of ALP. As shown in Figure 6A, Ag and exosome hybrid scaffold markedly increased ALP levels when compared with the control group. Accordingly, the ALP staining result was much more obvious in cells treated with the hybrid scaffolds as compared with the control (Figure 6B). The ALP activity result also revealed that the Ag and exosome hybrid scaffold markedly increased the ALP activity to the highest ALP level (Figure 6C). Previous studies indicated the important regulatory role of MSC-derived exosomes on tissue regeneration, which makes MSC-derived exosomes a promising modifying agent to immobilize various scaffolds, including tricalcium phosphate (β-TCP) (Zhang et al., 2016b) and titanium (Ti) surfaces (Wang et al., 2020). In this study, we also validated that MSC-derived exosomes may serve as natural regulators for promoting hBMSC differentiation. The mRNA levels of osteogenic markers (*ALP*, *OPN*, *Col-I,* and *Runx2*) were detected by RT-PCR. As shown in Figure 6D, we observed a significant increase in the expression of ALP, OCN, Col-I, and Runx2 in cells treated with the Ag and exosome hybrid scaffold. Previous study indicated that Ag nanoparticles at concentrations of 2.5 and 5 μg/ml were found to increase the osteogenic differentiation and mineralization of MSCs via autophagy regulation (He et al., 2020). It has also been demonstrated that osteogenic differentiation of MSCs was enhanced when exposed to 20 μM Ag nanoparticles at the size of 10 nm. Moreover, Ag nanoparticle–incorporated collagen scaffold could boost bone regeneration and induce early closure of the fracture gap *in vivo* (Zhang et al., 2015). In our study, we also observed that low dosage of Ag nanoparticle–incorporated PCL scaffold could enhance the osteogenic differentiation of hBMSCs. Together, the results demonstrated that MSC osteogenic differentiation was significantly increased in the exosome and Ag hybrid scaffold.

## Data Availability

The original contributions presented in the study are included in the article/supplementary material, and further inquiries can be directed to the corresponding authors.
